# Serum adipocytokine profile and metabolic syndrome in young adult female dermatomyositis patients

**DOI:** 10.6061/clinics/2016(12)06

**Published:** 2016-12

**Authors:** Marilda Guimarães Silva, Eduardo Ferreira Borba, Suzana Beatriz Veríssimo de Mello, Samuel Katsuyuki Shinjo

**Affiliations:** Faculdade de Medicina da Universidade de São Paulo, Clínica Médica, Reumatologia, São Paulo/SP, Brazil

**Keywords:** Adipocytokines, Cytokines, Dermatomyositis, Metabolic Syndrome, Myositis

## Abstract

**OBJECTIVES:**

To analyse the frequency of metabolic syndrome in young adult female dermatomyositis patients and its possible association with clinical and laboratory dermatomyositis-related features and serum adipocytokines.

**METHOD:**

This cross-sectional study included 35 dermatomyositis patients and 48 healthy controls. Metabolic syndrome was defined according to the 2009 Joint Interim Statement.

**RESULTS:**

Patient age was comparable in the dermatomyositis and control groups, and the median disease duration was 1.0 year. An increased prevalence of metabolic syndrome was detected in the dermatomyositis group (34.3% *vs*. 6.3%; *p*=0.001). In addition, increased serum adiponectin and resistin levels were noted in contrast to lower leptin levels. In dermatomyositis patients, adipocytokine levels were correlated with the levels of total cholesterol, low-density cholesterol, triglycerides and muscle enzymes. A comparison of dermatomyositis patients with (n=12) and without (n=23) syndrome metabolic revealed that adipocytokine levels were also correlated with age, and that dermatomyositis patients with metabolic syndrome tended to have more disease activity despite similar adipocytokine levels.

**CONCLUSIONS:**

Metabolic syndrome is highly prevalent in young adult female dermatomyositis patients and is related to age and disease activity. Moreover, increased serum adiponectin and resistin levels were detected in dermatomyositis patients, but lower serum leptin levels were observed.

## INTRODUCTION

Adipose tissue is an endocrine organ that influences body weight, glucose metabolism and lipid homeostasis 1,2, thus affecting several parameters of metabolic syndrome (MetS). In fact, MetS is characterized by an accumulation of conditions that include central obesity, dyslipidaemia, arterial hypertension and impaired glucose tolerance 3,4, which may be associated with inflammatory status 5. The endocrine influence of adipose tissue is determined by a group of active peptides known as adipocytokines, which can be characterized as having pro-inflammatory (e.g., leptin and resistin) or anti-inflammatory (e.g., adiponectin) effects 6.

Adipocytokines have been described in different systemic autoimmune diseases, such as systemic lupus erythaematosus 7,8 and rheumatoid arthritis 9,10, but they have rarely been studied in dermatomyositis (DM) 11,12. In one study, similar serum adiponectin levels were detected in DM patients and a control group, and the levels were related to pulmonary involvement 11. In another report, high levels of serum resistin were associated with DM inflammation, muscle injury and an increased global disease activity index 12. In addition to these findings, only two previous studies have assessed MetS and the risk factors of cardiovascular diseases (CVD) in adult patients with idiopathic inflammatory myopathies 13,14. Of note, de Moraes et al. 13 observed a high frequency of MetS (41.7%) in their DM cohort, likely due to the inclusion of older patients, both genders and postmenopausal women 13.

Thus, the present study aimed to evaluate the frequency of MetS specifically in a cohort of young female DM patients and to analyse the role of serum adipocytokines and their association with clinical and laboratory data, DM disease status and different aspects of MetS.

## MATERIALS AND METHODS

This cross-sectional study was performed at a single centre and included 35 consecutive female DM patients (age ≥18 and ≤45 years) enrolled from January 2012 to July 2014 who fulfilled all of the Bohan and Peter criteria items 15 and were regularly examined at our myopathy unit. Patients with clinically amyopathic DM, cancer-associated myositis, acute and/or chronic infections, liver and renal diseases, menopause or a body mass index (BMI) ≥30 kg/m^2^ were excluded. Forty-eight age-, gender- and BMI-matched healthy volunteers were recruited as a control group during the same period. The local Ethics Committee approved the study.

All participants underwent a clinical evaluation that included a standardized interview, and charts were extensively reviewed.

Demographic data included current age, household income status 16, gender, ethnicity, waist circumference, weight and BMI [weight/height^2^ (kg/m^2^)]. Clinical and laboratory data included age at disease onset, disease duration and serum levels of creatine phosphokinase, aldolase, alanine aminotransferase, aspartate aminotransferase, lactate dehydrogenase, fasting blood glucose, total cholesterol, high-density (HDL) cholesterol, low-density (LDL) cholesterol and triglycerides. These blood lipids were assessed in fasting serum samples by spectrophotometry.

Disease status was evaluated by the following questionnaires and scores: global assessment of the disease (by the physician and the patient) through the visual analogue scale (VAS) 17,18, Manual Muscle Testing (MMT-8) 19,20, Health Assessment Quality (HAQ) 19 and Myositis Disease Activity Assessment Visual Analogue Scales (MYOACT) 17.

Therapy data included the use of immunosuppressives, immunomodulatory corticosteroids [current dose and cumulative dose (since disease symptoms began)] and antimalarials.

The evaluation of comorbidities included the presence of arterial hypertension, dyslipidaemia, type 2 diabetes mellitus, hypothyroidism, myocardium infarction and ischaemic stroke. Dyslipidaemia was defined as having values of plasma total cholesterol >200 mg/dL, HDL cholesterol <40 mg/dL, LDL cholesterol >130 mg/dL, triglycerides >150 mg/dL or drug treatment for elevated LDL cholesterol or triglycerides 20.

Lifestyle evaluations of tobacco use, alcohol use, sedentary lifestyle 21 and food habit alterations were also conducted.

Family history of CVD was evaluated, including myocardial infarction, angina or sudden death in first-degree relatives before age 65 for women.

Laboratory evaluation: A blood sample (5 mL of blood) obtained from each participant after a 12-hour overnight fast was collected and immediately (<30 min) centrifuged at 3000 rpm for 10 minutes at 4°C. The serum was stored at -80°C until cytokine analysis (adiponectin, leptin and resistin) was performed using the Luminex 200- xMAP Instrument (Millipore, USA), as described elsewhere 22.

MetS was defined according to the 2009 Joint Interim Statement (JIS) of the International Diabetes Federation, National Heart, Lung, and Blood Institute, American Heart Association, World Heart Federation, International Atherosclerosis Society and International Association for the Study of Obesity 23. MetS was defined for DM patients and controls according to the presence of three or more of the following criteria: increased waist circumference (≥80 cm for South American women); increased triglycerides (≥150 mg/dL) (drug treatment for elevated triglycerides was an alternate indicator); reduced HDL-cholesterol (<50 mg/dL) (drug treatment for reduced HDL cholesterol was an alternate indicator); increased blood pressure (systolic pressure ≥130 mmHg and/or diastolic ≥85 mmHg) (antihypertensive drug treatment in patients with a history of hypertension was an alternative indicator); and increased fasting glucose (≥100 mg/d) (drug treatment of increased glucose was an alternate indicator).

CVD and its risk factors included the presence of coronary heart disease, cerebrovascular disease (for example, ischaemic stroke), deep vein thrombosis and pulmonary embolism, systemic arterial hypertension, diabetes mellitus, smoking, sedentary lifestyle, alcohol consumption and/or dyslipidaemia.

Statistical analysis: The Kolmogorov-Smirnov test was used to evaluate the distribution of each parameter. The demographic and clinical features were expressed as the means±standard deviations (SD) for continuous variables or as frequencies and percentages (%) for categorical variables. The median (25^th^-75^th^ interquartile range) was calculated for continuous variables that were non-normally distributed. Comparisons between the patient and control parameters were made using Student’s *t*-test or the Mann-Whitney test for continuous variables, and the chi-squared test or Fisher’s exact test were used to evaluate categorical variables. The correlations among the parameters were analysed by Spearman correlation. All analyses were performed using SPSS 15.0 statistical software (Chicago, USA). A value of *p*<0.05 was considered to indicate statistical significance.

## RESULTS

Thirty-five DM patients and 48 controls were evaluated. The mean age, BMI and weight and the frequencies of ethnicities and socioeconomic status were comparable between both groups (Table 1). The mean age at disease onset was 28.4 years, with a five-month period of symptoms prior to diagnosis and a median disease duration of 1.0 year. As expected, all muscle enzymes were significantly increased in DM patients. The main disease status parameters are presented in Table 1.

Two-thirds of the DM patients were using therapy, with a total cumulative dose of prednisone of 15.40 g. Eight patients (22.9%) were also using an antimalarial [chloroquine diphosphate (250 mg/day) or hydroxychloroquine sulphate (400 mg/day)]. Approximately half of the patients were using at least one immunosuppressive or immunomodulatory drug: azathioprine (2-3 mg/kg/day), methotrexate (15-25 mg/week), cyclosporine (1.5-2.5 mg/kg/day), mycophenolate mofetil (2-3 g/day), rituximab [1 g, intravenous, at baseline and after one month (first cycle) and this schema was repeated after six months], cyclophosphamide (0.8 g/m^2^ body surface), leflunomide (20 mg/day) and/or intravenous human immunoglobulin (2 g/kg, daily, two consecutive days) (Table 1).

An increased frequency of MetS was detected in DM patients compared to controls (34.3% *vs*. 6.3%; *p*=0.001) (Table 2). The abdominal circumference, which is a part of the MetS criteria, was also increased in DM patients compared to the control group (*p*<0.001).

No difference was observed between groups regarding comorbidities, lifestyle or family history of CVD, with the exception of diabetes mellitus [present only in DM patients (2.9%)] and tobacco use [present only in the control group (6.3%)]. No cases of ischaemic stroke, myocardial infarction or alcohol consumption were included in either group.

Regarding the laboratory parameters, both groups had similar fasting blood glucose, total cholesterol and LDL cholesterol levels. However, higher serum levels of HDL cholesterol and leptin were detected in controls, whereas higher serum levels of LDL cholesterol, triglycerides, insulin, adiponectin and resistin levels were identified in DM patients.

A further analysis of DM patients revealed that the serum level of adiponectin was moderately correlated with the levels of total cholesterol (rho=0.444; *p*=0.014) and LDL cholesterol (rho=0.443; *p*=0.014) and that the level of resistin was correlated with that of triglycerides (rho=0.400; *p*=0.029). The leptin level was negatively associated with those of aldolase (rho=-0.502; *p*=0.008), triglycerides (rho=-0.448; *p*=0.017), aspartate aminotransferase (rho=-0.570; *p*=0.002) and alanine aminotransferase (rho=-0.582; *p*=0.001). Importantly, these cytokines were not correlated with other parameters.

A comparison of DM patients with (n=12) or without (n=23) MetS revealed that patients with MetS were significantly older (Table 3).

The distributions of ethnicity, household income status, age at disease onset, duration between diagnosis and symptoms, disease duration, BMI, weight, treatment (corticosteroid, immunosuppressives, immunomodulatory and/or antimalarial) and comorbidities were similar in DM patients with or without MetS.

The heliotrope and Gottron’s signal distributions were also similar in both groups in contrast to the “V” sign and “shawl” sign, which were more frequent in the group with MetS. In this group, the disease status parameters were also increased, with the exception of the HAQ and MYOACT scores, which were similar.

The insulin and cytokine (adiponectin, leptin and resistin) parameters were also comparable between DM patients with and without MetS.

## DISCUSSION

The present study revealed an increased prevalence of MetS in young adult female DM patients. Moreover, higher serum levels of adiponectin and resistin associated with lower leptin levels were observed in DM patients with MetS. Importantly, the presence of MetS was associated with age and current disease status parameters.

A great advantage of the present study was that it evaluated young premenopausal female patients with well-defined DM. Furthermore, MetS was defined according to current criteria 22, which are now accepted worldwide.

Previous studies have reported a high prevalence of MetS in several rheumatic diseases that ranged from 14 to 62.8% 13,14,24,25. However, only two of these studies analysed the prevalence of MetS in idiopathic inflammatory myopathies 13,14], and both used the ATP III criteria 4. These studies identified MetS in 41.7% and 45.7% of patients with DM and PM, respectively 13,14. However, it should be emphasized that these studies evaluated both genders and included postmenopausal women because they had a large range of ages. In addition, the patients’ BMIs were not matched with control groups, which could have contributed to the high frequency of MetS in these samples.

Another important point in the present study was the exclusion of all possible confounding parameters that could have interfered with our analysis. However, we observed a high frequency of MetS in DM patients (34.3%) compared to healthy individuals (6.3%). Although the patient group was BMI-matched to the healthy individual group, we observed that the former had a larger waist circumference, which seemed to be the most important contributing factor to MetS in these patients.

Concerning comorbidities, a low prevalence of risk factors for CVD was observed herein in contrast to a previous study 13, which could be explained by the inclusion of young adults with a short time of disease duration.

In the present study, we observed a high prevalence of dyslipidaemia in our patients, similar to previous studies 13,26,27. Of note, this dyslipidaemia may be correlated with disease activity.

A further analysis of DM patients revealed higher serum levels of adiponectin and resistin in contrast to lower serum levels of leptin. Adiponectin, which is a hormone that is abundantly secreted by adipocytes under normal conditions, has anti-diabetic, anti-inflammatory and anti-atherogenic properties 6,28,29. However, this cytokine can be induced in skeletal muscle after an inflammatory condition or metabolic/oxidative stress 30-32. In this regard, the higher serum adiponectin levels observed in DM patients could be a protective mechanism to counterbalance the chronic, systemic, inflammatory and immunological processes of this disease.

Interestingly, the adiponectin levels in DM patients were associated with their total cholesterol and LDL cholesterol levels but not with other parameters, including insulinaemia and classical MetS. In this regard, waist circumference, which is related to visceral obesity, was significantly increased in DM patients. However, it was not associated with adiponectin levels. Therefore, the high rate of adiponectinaemia could not be explained by possible altered fat mass or distribution. Moreover, the corticosteroid therapy did not appear to have an effect on adiponectin values because the groups received similar corticosteroid doses.

In contrast to adiponectin, resistin is considered a pro-inflammatory protein with atherogenic effects and is associated with peripheral insulin resistance 30,33. A previous study showed that high serum resistin levels in patients with inflammatory myopathies (DM and other myositis) were associated with inflammation, muscle damage and an increased global disease activity index 12. These authors and others showed that resistin gene expression in muscle tissue was significantly increased in patients compared to controls 12,34. Thus, resistin could play a potential role in the pathogenesis of inflammatory myopathies. This possibility was confirmed in our study because resistin was also increased in DM patients, but no correlation was detected with clinical and laboratory parameters.

In contrast, we observed low serum levels of leptin in our patients with DM. Leptin is a cytokine that is produced mainly by adipocytes and participates in a wide variety of physiological processes, including modulating immune responses by acting as a pro-inflammatory cytokine 35. Paradoxically, in the present study, leptin was negatively associated with serum levels of muscle enzymes and triglycerides.

The comparison of DM patients with and without MetS revealed that this condition is related to ageing, similar to a previous study (in a univariate analysis) 13. Moreover, MetS in DM patients was not correlated with corticosteroid use, reinforcing the data of the previous study 13.

Similarly, the presence of cutaneous lesions (“V” sign and “shawl” sign) was more frequently observed in patients with MetS, which should be confirmed in other studies.

The present study had some limitations. The cross-sectional design, rather than a longitudinal analysis, limited the evaluation of MetS parameters and variation in adipocytokines. In addition, we must consider the possible inclusion of a more severe disease due to the characteristics of our tertiary care centre.

In summary, a high frequency of MetS was observed in young DM patients, which was associated with high adiponectin and resistin levels and low leptin levels. In addition, MetS occurred predominantly due to the presence of abdominal abnormalities and was associated with ageing and disease activity status. These data reinforce the role of adipokines and provide a better understanding of their mechanisms and regulatory pathways.

## AUTHOR CONTRIBUTIONS

Silva MG and Shinjo SK were responsible for the study design, included cases, material analysis, statistics and manuscript writing. Borba EF and Mello SB were responsible for the manuscript writing.

## Figures and Tables

**Table 1:** Demographic, Clinical and Laboratory Features of Dermatomyositis Patients and Healthy Individuals.

Parameters	DM (n=35)	Control (n=48)	*P*
Age (years)	33.3±7.6	33.2±6.5	0.951
White ethnicity	26 (74.3)	34 (70.8)	0.807
Body mass index (kg/m^2^)	24.5±3.2	24.3±2.6	0.872
Weight (kg)	63.5±9.3	63.7±7.4	0.914
Socioeconomic status	31 (81.6)	46 (95.8)	0.586
Age at disease onset (years)	28.4±9.0	-	-
Duration: diagnosis - symptoms (months)	5 (2-18)	-	-
Disease duration (years)	1.0 (0-6.0)	-	-
Creatine phosphokinase (U/L)	124 (86-458)	98 (72-122)	0.011
Aldolase (U/L)	6.3 (4.0-11.0)	3.4 (2.8-3.4)	<0.001
Lactic dehydrogenase (U/L)	423 (654-715)	322 (293-385)	0.001
Alanine aminotransferase (U/L)	22 (13-51)	15 (12-20)	0.003
Aspartate aminotransferase (U/L)	19 (16-71)	19 (16-21)	0.001
MMT-8 score (0-80)	76 (70-80)	-	-
HAQ score (0.00-3.00)	0.86 (0.00-0.71)	-	-
Patient VAS (0-10)	3 (0-5)	-	-
Physician VAS (0-10)	4 (0-5)	-	-
MYOACT	0 (0-1)	-	-
Prednisolone			-
Current use	23 (65.7)	-	-
Total cumulative dose (g)*	15.40 (5.90-27.69)		
Antimalarial	8 (22.9)	-	-
IS/IM**			
None	17 (48.6)	-	-
One	12 (34.3)	-	-
Azathioprine	6 (17.1)		
Methotrexate	3 (8.6)		
Cyclosporine	2 (5.7)		
Cyclophosphamide	1 (2.8)		
Two	6 (17.1)	-	-
Methotrexate + azathioprine	1 (2.8)		
Methotrexate + leflunomide	1 (2.8)		
Methotrexate + cyclosporine	1 (2.8)		
Methotrexate + IVIg	1 (2.8)		
Cyclosporine + azathioprine	1 (2.8)		
Mycophenolate mofetil + rituximab	1 (2.8)		

Results expressed as percentages (%), means ± standard deviation, or medians (25^th^ - 75^th^ interquartile range).

DM: dermatomyositis; IS: immunosuppressive drugs; IM: immunomodulatory drugs; IVIg: intravenous human immunoglobulin; MYOACT: myositis disease activity assessment visual analogue scales.

* Since disease symptoms began;

** Azathioprine (2-3 mg/kg/day), methotrexate (15-25 mg/week), cyclosporine (1.5-2.5 mg/kg/day), mycophenolate mofetil (2-3 g/day), rituximab [1 g, intravenous, at baseline and after one month (first cycle) and this schema was repeated after six months], cyclophosphamide (0.8 g/m^2^ body surface), leflunomide (20 mg/day) and/or intravenous human immunoglobulin (2 g/kg, 1x/day, two consecutive days).

**Table 2:** Metabolic Syndrome and Laboratory Parameters of Patients with Dermatomyositis and Healthy Individuals.

Parameters	DM (n=35)	Control (n=48)	*P*
Metabolic syndrome	12 (34.3)	3 (6.3)	0.001
Abdominal circumference (cm)	88.6±10.1	78.9±9.0	<0.001
≥80 cm	26 (74.3)	22 (45.8)	0.013
Systemic arterial hypertension	3 (8.6)	1 (2.1)	0.305
Fasting blood glucose (mg/dL)	81 (76-89)	79 (70-84)	0.157
≥100 mg/dL	5 (14.3)	1 (2.1)	0.078
Total cholesterol level (mg/dL)	175.2±36.8	185.4±31.5	0.190
LDL cholesterol level (mg/dL)	103.2±32.1	107.03±27.0	0.540
HDL cholesterol level (mg/dL)	48 (42-63)	55 (52-65)	0.017
≤50 (mg/dL)	18 (51.4)	7 (14.6)	0.001
Triglyceride level (mg/dL)	83 (61-180)	82 (64-109)	0.017
≥150 (mg/dL)	13 (37.1)	4 (8.3)	0.002
Insulin (U/L)	13.0 (9.3-19.5)	7.5 (5.4-12.1)	<0.001
Adiponectin (ng/mL)	87.3 (56.1-115.8)	58.8 (39.5-75.8)	0.010
Leptin (ng/mL)	7.9 (0.7-13.6)	14.8 (7.9-22.1)	0.004
Resistin (pg/mL)	100 (80-167)	89 (70-112)	0.049

Results expressed as percentages (%) or medians [25^th^ - 75^th^ interquartile range]. CVD: cardiovascular disease; DM: dermatomyositis; HDL: high-density cholesterol; LDL: low-density cholesterol.

**Table 3:** Comparison of DM Patients with and Without Metabolic Syndrome.

Parameters	MetS (+) (n=12)	MetS (-) (n=23)	*P*
Age (years)	36.7±5.6	31.5±8.0	0.035
White ethnicity	7 (58.3)	19 (82.6)	0.220
Socioeconomic status	12 (100.0)	19 (82.6)	0.536
Age at disease onset (years)	31.0±10.0	27.1±8.3	0.260
Duration: diagnosis - symptoms (months)	4 (1-14)	5 (3-18)	0.381
Disease duration (years)	0 (0-9)	2 (0-6)	0.362
Body mass index (kg/m^2^)	25.0±2.6	24.2±3.5	0.511
Weight (kg)	63.6±9.7	63.4±9.2	0.938
Cutaneous manifestations			
Heliotrope	10 (83.3)	20 (87.0)	1.000
Gottron’s signal	12 (100.0)	21 (91.3)	0.536
“V” sign	6 (50.0)	3 (13.0)	0.038
“Shawl” sign	5 (41.7)	1 (4.3)	0.012
Lipodystrophy	0	0	1.000
Calcinosis	0	0	1.000
MMT-8 score (0-80)	66 (60-76)	80 (74-80)	0.002
HAQ score (0.00-3.00)	1.15 (0.00-2.68)	0.57 (0.00-1.71)	0.172
Patient VAS (0-10)	6 (4-7)	2 (0-5)	0.001
Physician VAS (0-10)	6 (4-7)	2 (0-5)	0.011
MYOACT	0 (0-4)	0 (0-0)	0.344
Creatine phosphokinase (U/L)	122 (71-1447)	130 (84-232)	0.817
Aldolase (U/L)	6.9 (5.3-31.4)	5.7 (3.8-10.3)	0.292
Lactic dehydrogenase (U/L)	659 (425-1170)	372 (282-476)	0.010
Alanine aminotransferase (U/L)	57 (65-143)	20 (16-32)	0.028
Aspartate aminotransferase (U/L)	56 (22-96)	19 (19-30)	0.031
Prednisolone			
Current use	9 (75.0)	14 (60.9)	0.476
Cumulative dose* (g)	7.1 (1.6-23.0)	15.6 (11.0-30.4)	0.156
Antimalarials	2 (16.7)	6 (26.1)	0.685
IS/IM**			
None	9 (75.0)	9 (39.1)	0.075
One	2 (16.7)	10 (43.5)	0.149
Two	1 (8.3)	4 (17.4)	0.640
Systemic arterial hypertension	3 (25.0)	0	-
Abdominal circumference (cm)	92.3±9.7	86.5±9.9	0.112
≥80 cm	10 (83.3)	16 (69.6)	0.450
Diabetes mellitus	1 (8.3)	0	-
Ischaemic stroke	0	0	-
Myocardial infarction	0	0	-
Hypothyroidism	2 (16.7)	1 (4.3)	0.266
Sedentary lifestyle	1 (8.3)	2 (8.7)	1.000
Food habit alterations	1 (8.3)	1 (4.3)	1.000
Alcohol consumption	0	0	-
Tobacco	0	0	-
Family history of CVD	1 (8.3)	0	-
Fasting blood glucose (mg/dL)	82 (65-144)	80 (74-85)	0.526
≥100 mg/dL	4 (33.3)	1 (4.3)	0.038
Total cholesterol level (mg/dL)	179 (145-212)	165 (144-191)	0.668
LDL cholesterol level (mg/dL)	106.8±33.8	101.3±31.8	0.652
HDL cholesterol level (mg/dL)	39.2±11.0	59.3±18.2	<0.001
≤50 (mg/dL)	11 (91.7)	7 (30.4)	0.001
Triglycerides level (mg/dL)	160 (99-241)	80 (60-136)	0.016
≥150 (mg/dL)	9 (75.0)	4 (17.4)	0.002
Insulin (U/L)	16.5 (9.4-30.0)	12.3 (6.8-18.2)	0.321
Adiponectin (ng/mL)	74.8 (39.3-125.0)	95.1 (54.0-116.3)	0.349
Leptin (ng/mL)	4.3 (39.3-125.1)	8.9 (0.7-14.7)	0.709
Resistin (pg/mL)	204 (86-292)	92 (76-123)	0.077

Results expressed as percentages (%), means ± standard deviation, or medians (25^th^ - 75^th^ interquartile range).

CVD: cardiovascular disease; DM: dermatomyositis; HDL: high-density cholesterol; IS: immunosuppressive drugs; IM: immunomodulatory drugs; LDL: low-density cholesterol; MYOACT: myositis disease activity assessment visual analogue scales.

* Since disease symptoms began;

* Azathioprine (2-3 mg/kg/day), methotrexate (15-25 mg/week), cyclosporine (1.5-2.5 mg/kg/day), mycophenolate mofetil (2-3 g/day), rituximab [1 g, intravenous, at baseline and after one month (first cycle) and this schema was repeated after six months], cyclophosphamide (0.8 g/m^2^ body surface), leflunomide (20 mg/day) and/or intravenous human immunoglobulin (2 g/kg, 1x/day, two consecutive days).
